# Cochlear supporting cells function as macrophage-like cells and protect audiosensory receptor hair cells from pathogens

**DOI:** 10.1038/s41598-020-63654-9

**Published:** 2020-04-21

**Authors:** Yushi Hayashi, Hidenori Suzuki, Wataru Nakajima, Ikuno Uehara, Atsuko Tanimura, Toshiki Himeda, Satoshi Koike, Tatsuya Katsuno, Shin-ichiro Kitajiri, Naoto Koyanagi, Yasushi Kawaguchi, Koji Onomoto, Hiroki Kato, Mitsutoshi Yoneyama, Takashi Fujita, Nobuyuki Tanaka

**Affiliations:** 1 Department of Molecular Oncology, Institute for Advanced Medical Sciences, Nippon Medical School, Tokyo, Japan; 2 Division of Morphological and Biomolecular Research, Nippon Medical School, Tokyo, Japan; 3 Department of Microbiology, Kanazawa Medical University School of Medicine, Ishikawa, Japan; 4 Neurovirology Project, Tokyo Metropolitan Institute of Medical Science, Tokyo, Japan; 5 Department of Otolaryngology, Head and Neck Surgery, Kyoto University, Kyoto, Japan; 6 Division of Molecular Virology, Department of Microbiology and Immunology, The Institute of Medical Science, The University of Tokyo, Tokyo, Japan; 7 Division of Molecular Immunology, Medical Mycology Research Center, Chiba University, Chiba, Japan; 8 Laboratory of Molecular Genetics, Institute for Virus Research, Kyoto University, Kyoto, Japan

**Keywords:** Adaptive immunity, Antimicrobial responses, Sensory systems

## Abstract

To protect the audiosensory organ from tissue damage from the immune system, the inner ear is separated from the circulating immune system by the blood-labyrinth barrier, which was previously considered an immune-privileged site. Recent studies have shown that macrophages are distributed in the cochlea, especially in the spiral ligament, spiral ganglion, and stria vascularis; however, the direct pathogen defence mechanism used by audiosensory receptor hair cells (HCs) has remained obscure. Here, we show that HCs are protected from pathogens by surrounding accessory supporting cells (SCs) and greater epithelial ridge (GER or Kölliker’s organ) cells (GERCs). In isolated murine cochlear sensory epithelium, we established Theiler’s murine encephalomyelitis virus, which infected the SCs and GERCs, but very few HCs. The virus-infected SCs produced interferon (IFN)-α/β, and the viruses efficiently infected the HCs in the IFN-α/β receptor-null sensory epithelium. Interestingly, the virus-infected SCs and GERCs expressed macrophage marker proteins and were eliminated from the cell layer by cell detachment. Moreover, lipopolysaccharide induced phagocytosis of the SCs without cell detachment, and the SCs phagocytosed the bacteria. These results reveal that SCs function as macrophage-like cells, protect adjacent HCs from pathogens, and provide a novel anti-infection inner ear immune system.

## Introduction

The inner ear was previously regarded as an immune-privileged site because the blood-labyrinthine barrier prevents the peripheral immune system accessing this site^1^. To minimize any collateral tissue damage induced by an immune reaction, delicate tissues, such as those of the central nervous system (CNS), sensory organs (eyes and ears) and gonads (testes and ovaries), are separated from the peripheral blood by a physical barrier^2^. In the mammalian inner ear, audiosensory receptor hair cells (HCs) are located over the basilar membrane in the organ of Corti, which vibrates in response to sound waves^3^. This sensory organ lies in the cochlear duct (known as the scala media) and is separated from the vestibular duct and the tympanic duct by the avascular Reissner’s membrane and a basilar membrane, respectively^4^. To offset the constant auditory sensation of heartbeats, the direct blood supply to the organ of Corti is sparse. The oxygen supply to HCs is provided by the dense capillary network of the stria vascularis, which is present in the lateral wall of the cochlear duct, constitutes the major feeding vessels^1,5^.

HCs transduce the mechanical force generated by sound waves into electrical signals^6^ and are aligned in four rows, one of which, the inner HCs (IHCs) detect sound, while the other three rows of outer HCs (OHCs) have amplitude and frequency-resolving capabilities^3,6^. OHCs are surrounded by supporting cells (SCs), termed Hensen’s cells and Claudius’ cells^3^. IHCs are surrounded by greater epithelial ridge (GER or Kölliker’s organ) cells (GERCs) in the immature neonatal inner ear, which develop later on into mature SCs and are important for HC development^7,8^. SCs are linked to each other and to HCs via tight and adherent junctions; they form rigid cytoskeletons and maintain the structural integrity of the audiosensory organs during sound stimulation and head movement^6^. Although SCs have several functions in the cochlea related to gap junctions containing connexin proteins including K^+^-recycling, nutrient and energy supply, and generation and maintenance of the unique electrochemical environments of the endolymph and perilymph^9^, their roles in the immune system have not been elucidated.

Macrophages and leukocytes are present in the cochlea of the avian inner ear^10^, especially within the hyaline/cuboidal cell region, which is located just below the sensory epithelium and near the basilar membrane^11^. A recent study reported that ionized calcium-binding adapter molecule 1 (Iba1)-positive resident macrophages were present in high numbers in the spiral ligament, spiral ganglion, and stria vascularis of the postnatal cochlea in mice^12^. Moreover, another study using mice with macrophages expressing green fluorescent protein (GFP) showed that very few macrophages (0–4/100 μm) were present in sensory epithelium, and that selective HC lesions increased the number of macrophages, which might have migrated from under the basilar membrane^13^. This suggests that macrophages migrate from outside the sensory epithelium in response to HC damage. In addition, macrophages accumulated around the nerve fibres at the habenula perforata, where nerves enter and exit the organ of Corti^14^, suggesting macrophages might enter from the habenular opening. In the stria vascularis, which supplies oxygen to the HCs^15^, vascular endothelial cells, pericytes and perivascular-resident macrophage-like melanocytes (PVM/Ms) form a blood-labyrinthine barrier^1^. For PVM/Ms, recent findings suggested that perivascular macrophages require more careful analysis to have melanocyte characteristics^16^, but these cells are critical regulatory cells in the fluid-blood barrier and may form the first line of immunological defence^17^. These cells were reported to inhibit pathogen entry via the stria vascularis by regulating the fluid-blood barrier, similar to what occurs in the CNS, and to function as macrophages that protect against pathogens^1,18^. A recent study using bone marrow chimeric mice showed that macrophages are present in the spiral ligament and spiral ganglion, but not in the sensory epithelium^19^. This suggests that there is a mechanism to prevent infectious agents such as viruses entering the organ of Corti. However, the defence mechanism around HCs when pathogens invade beyond these lines of defence awaits elucidation.

One of the earliest innate antiviral defence mechanisms is the type I interferon (IFN) system^20^. We previously found that Theiler’s murine encephalomyelitis virus (TMEV) infection in murine newborn cochlear sensory epithelium results in IFN-α/β production^21^. TMEV is a small RNA picornavirus commonly used as an experimental model system for blood-brain barrier disruption^22^. In the present study, we isolated murine newborn cochlear sensory epithelium and investigated the effects of viral infection on epithelial cells, because these cells can only be isolated from the relatively soft newborn temporal bone. Using this experimental system, we sought to identify whether a protection system against infection in the sensory epithelium of the organ of Corti exists.

## Results

### IFN-α/β produced by SCs and GERCs protects HCs against virus infection

**Figure 1:**
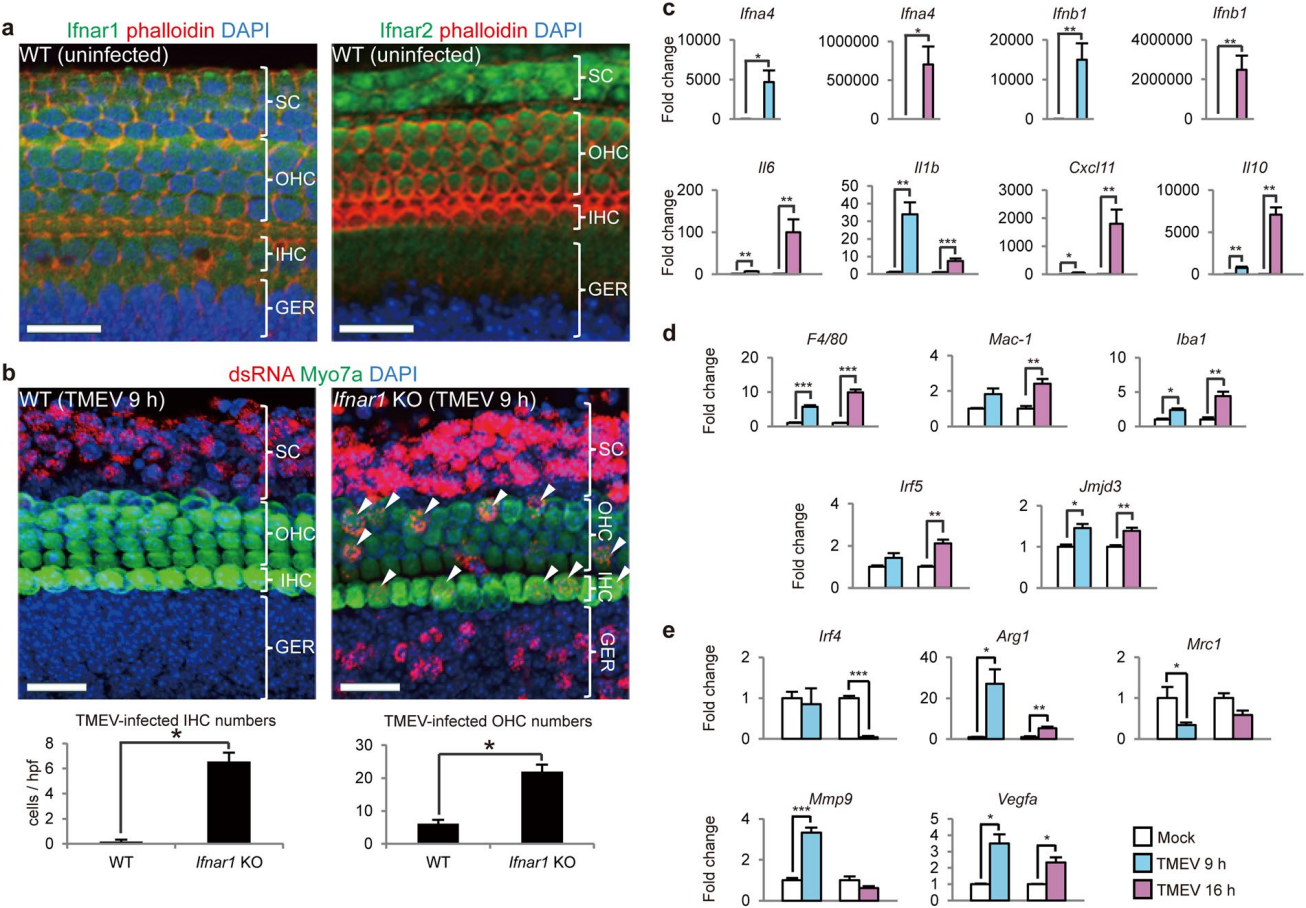
Type I IFN from SCs and GERCs protects HCs from virus infection. (**a**) Expression sites for Ifnar1 (green) and Ifnar2 (green). (**b**) Ifnar1-deficient mice (n = 7) had significantly more TMEV-infected HCs (arrowheads) than wildtype (WT) mice (n = 6) (*P < 0.0001, *t*-test). (**c**) Type I IFN (IFN alpha 4 (*Ifna4*), IFN beta 1 (*Ifnb1*)) (Mock: n = 5, TMEV: n = 5) and other inflammatory cytokines and chemokines, such as interleukin 6 (*Il6*), interleukin 1 beta (*Il1b*), chemokine *(C-X-C motif)* ligand 11 (*Cxcl11*) *(*Mock 9 h: n = 4, TMEV 9 h: n = 5, Mock 16 h: n = 6, TMEV 16 h: n = 5) and interleukin 10 (*Il10*) (Mock 9 h: n = 3, TMEV 9 h: n = 4, Mock 16 h: n = 3, TMEV 16 h: n = 4), were expressed during virus infection (*P < 0.05, **P < 0.01, ***P < 0.001, *t*-test). (**d**) qRT-PCR analysis showing upregulated macrophage markers (*F4/80*, *Mac-1*, and *Iba1*), and M1 (*Irf5*) and M2 (*Jmjd3*) macrophage markers (*P < 0.05, **P < 0.01, ***P < 0.001, *t*-test, Mock 9 h: n = 3, TMEV 9 h: n = 4, Mock 16 h: n = 3, TMEV 16 h: n = 4). (**e**) qRT-PCR analysis of M2 markers after TMEV infection, including IFN regulatory factor 4 (*Irf4*) (Mock 9 h: n = 3, TMEV 9 h: n = 4, Mock 16 h: n = 3, TMEV 16 h: n = 4), arginase 1 (*Arg1*) (Mock 9 h: n = 3, TMEV 9 h: n = 4, Mock 16 h: n = 6, TMEV 16 h: n = 5), mannose receptor C type 1 (*Mrc1*) (Mock 9 h: n = 3, TMEV 9 h: n = 4, Mock 16 h: n = 3, TMEV 16 h: n = 4), matrix metallopeptidase 9 (*Mmp9*) (Mock 9 h: n = 4, TMEV 9 h: n = 5, Mock 16 h: n = 6, TMEV 16 h: n = 5) and vascular endothelial growth factor A (*Vegfa*) (Mock 9 h: n = 3, TMEV 9 h: n = 4, Mock 16 h: n = 3, TMEV 16 h: n = 4) (*P < 0.05, **P < 0.01, ***P < 0.001, *t*-test). Scale bars, 20 µm. Error bars, standard errors.

As shown in Fig. 1a, the IFN (alpha and beta) receptor 1 (Ifnar1) and IFN (alpha and beta) receptor 2 (Ifnar2) subunits of IFN-α/β receptors^23^ were expressed in OHCs and IHCs. Infections with TMEV were mainly established in SCs, and infections in HCs were rarely observed from 9 h post-infection (Fig. 1b, left). Conversely, virus-infected HC numbers were seen to increase significantly in the *Ifnar1*-null^24^ sensory epithelium (Fig. 1b, right), demonstrating that the type I IFN system was present and functional in the studied sensory epithelium. The isolated sensory epithelium contained only HCs, SCs and GERCs, with no lateral wall containing stria vascularis, pericytes and PVM/Ms present^21^. Interestingly, expression of IFN-α/β and macrophage-associated marker mRNA was induced, suggesting the existence of macrophage-like cells in epithelial tissue (Fig. 1c). The macrophage markers *F4/80*, macrophage-1 antigen (*Mac-1*), *Iba1* and IFN regulatory factor 5 (*Irf5*) from M1 macrophages, and jumonji domain containing-3 (*Jmjd3*) and others from M2 macrophages^25–27^, were also induced by the viral infection (Fig. 1d,e).

### Virus-infected SCs and GERCs become detached from their cell layers

**Figure 2:**
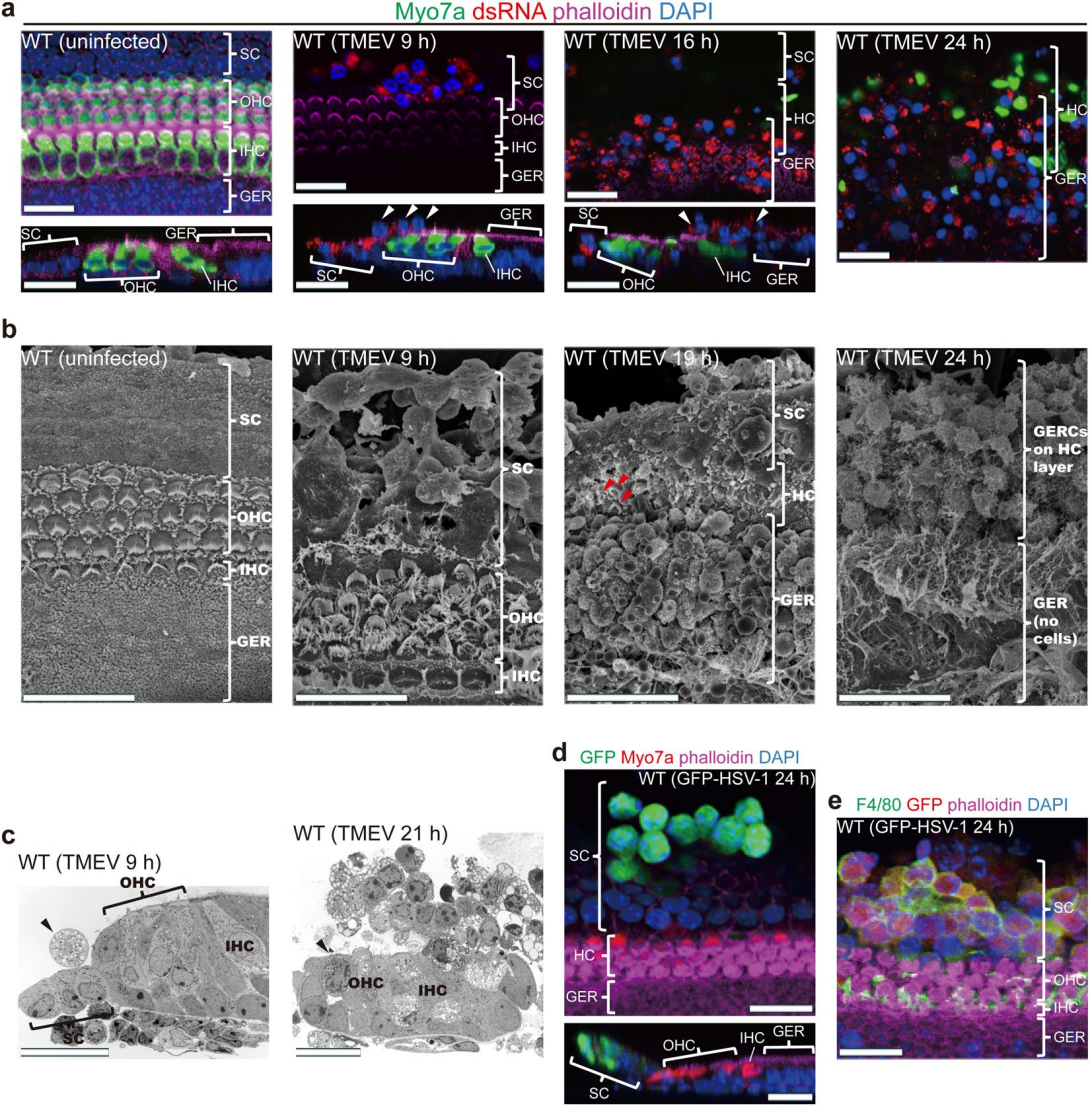
SCs and GERCs embedded and fixed in the cochlear sensory epithelium start to migrate to HCs upon virus infection. (**a–c**) Immunostaining (whole mounts and sections; **a**) and electron microscope (**b,c**) analysis of SCs/GERCs [white (**a**) and black (**c**) arrowheads] during TMEV infection. Red arrowheads in (**b**) indicate stereocilia survival in the HCs. Under steady-state conditions, all cells in the sensory epithelium were fixed and arranged as cobblestones. At 9–12 h after TMEV incubation, virus-infected SCs migrated to the epithelial surface and to HCs. At 16–21 h after TMEV infection, virus-infected GERCs also moved to the epithelium and migrated to the HC layer. At 24 h after TMEV infection, most of the HC layer was covered by TMEV-infected SCs/GERCs. (**d,e**) Immunostaining of SCs during GFP-HSV-1 infection. GFP-HSV-1-infected SCs moved to the epithelial surface (**d**) and expressed F4/80 (**e**), as observed during RNA virus infection. Immunostaining and TEM scale bars, 20 µm. SEM scale bars, 5 µm.

To identify which cells express macrophage-associated genes, we analysed the virus-infected sensory epithelium. The virus-infected SCs and GERCs became detached from their cell layers (Supplementary Videos 1 and 2). At 9 h post-infection with TMEV, several SCs left their cell layer and entered the HC layer (Fig. 2a; TMEV 9 h). Subsequently, the virus-infected GERCs also detached from their cell layer (Fig. 2a; TMEV 16 h and 24 h). The virus-infected sensory epithelia were then analysed by electron microscopy (Fig. 2b,c and Fig. S1a–c). Similar SC migration was also observed following infection with HSV-1, a major cause of virus-induced sudden sensorineural hearing loss (Fig. 2d,e). The ectopic expression of mitochondrial antiviral signalling (Mavs, also called IPS-1)^28^, a downstream signal activator of the retinoic acid inducible gene-I (RIG-I) and melanoma differentiation-associated gene 5 (MDA5), induced SC migration (Fig. S1d,e). These observations suggest that RIG-I-like receptors were able to detect the TMEV infection and induce the release of SCs from cell-cell adhesion constraints.

### Virus-infected SCs and GERCs each show an activated macrophage phenotype

**Figure 3:**
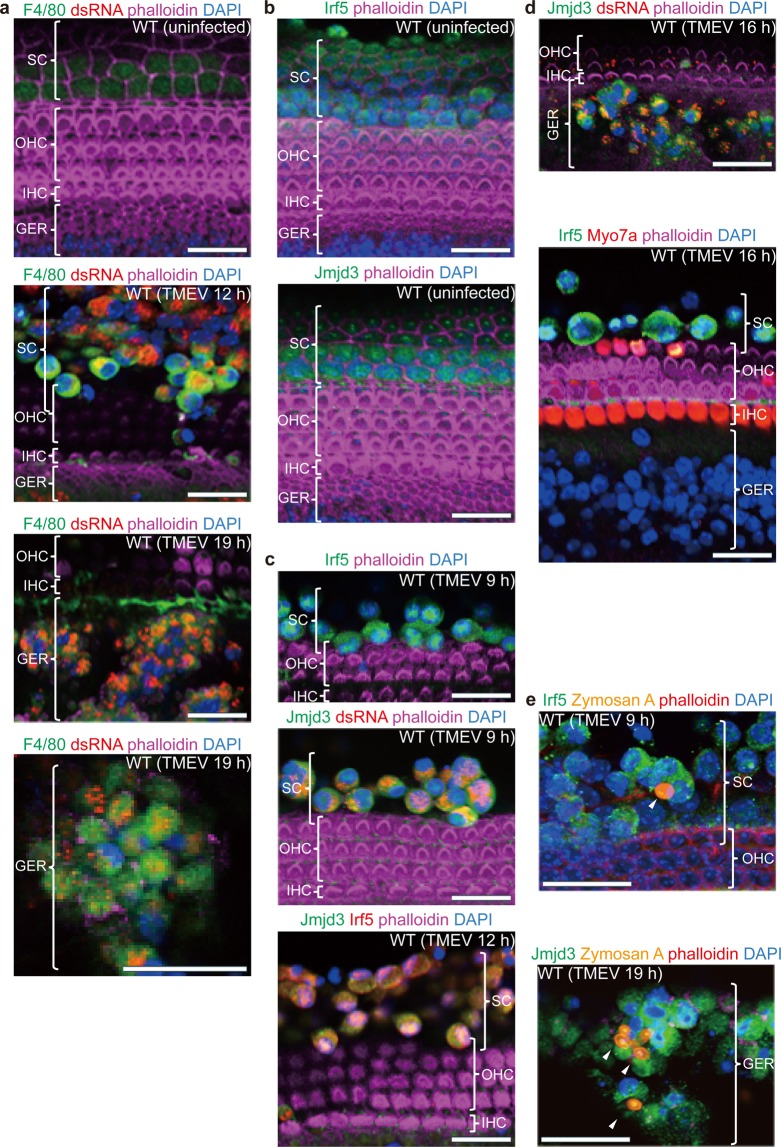
Macrophage marker upregulation in SCs and GERCs during TMEV infection. (**a**) Under steady-state conditions, F4/80 (green) was weakly expressed in SCs, but not in GERCs. Conversely, the SCs and GERCs migrating to the HCs during the virus infection exhibited strong F4/80 expression (green), indicating the virus-induced activation of SCs and GERCs as macrophages. (**b–d**) Under steady-state conditions, both Irf5 (green) (M1 marker) and Jmjd3 (green) (M2 marker) were expressed in SCs, but not in GERCs (**b**). Virus incubation increased the expression of Irf5 (green) and Jmjd3 (green) in the SCs, which was confirmed by double-positive (Jmjd3, green; Irf5, red) expression in SCs (**c**). The GERCs expressed Jmjd3 (green), but not Irf5 (green) during the virus infection (**d**). These findings indicate that virus-infected SCs exhibit both M1 and M2 characteristics, whereas virus-infected GERCs exhibit only M2 characteristics. (**e**) Virus-activated SCs and GERCs phagocytised *Saccharomyces cerevisiae* conjugated with Alexa Fluor 488 (white arrowheads), demonstrating the macrophage-like ability of these cell types. Scale bars, 20 µm.

In the absence of the TMEV infection, SCs, but not GERCs, expressed low levels of F4/80, but upon infection these levels were significantly enhanced or induced the expression of F4/80 in the SCs or GERCs, respectively (Fig. 3a). In response to the virus infection, enhanced expression of Irf5 and Jmjd3 was seen in the SCs (Fig. 3b,c), indicative of both proinflammatory M1 and anti-inflammatory M2 macrophage types^29^. Conversely, the GERCs expressed Jmjd3, but not Irf5, during the virus infection (Fig. 3d), indicative of an anti-inflammatory M2 macrophage phenotype^29^. Moreover, the expression of other macrophage markers was induced by the virus infection (Fig. S2), and both virus-infected SCs and GERCs alike exhibited phagocytosis (Fig. 3e), a hallmark of functional macrophages. This suggests that SCs and GERCs function similarly to the tissue-resident macrophages previously reported to regulate tissue homeostasis by acting as sentinels responding to physiological changes and pathogenic microorganisms^30,31^. Conversely, the SCs and GERCs were anchored by tight junctions in the absence of the TMEV infection. Similar to the inner ear, the blood-brain barrier prevents access of the peripheral immune system to the CNS, where microglial cells (resident macrophages), which arise from yolk sac macrophages during foetal development, regulate brain development, maintenance of neuronal networks, and injury repair. Moreover, microglia function as macrophages by eliminating dead cells, microbes and other pathogens that may damage the CNS^32,33^.

### The sensory epithelium’s immune system contains macrophage-like cells

**Figure 4:**
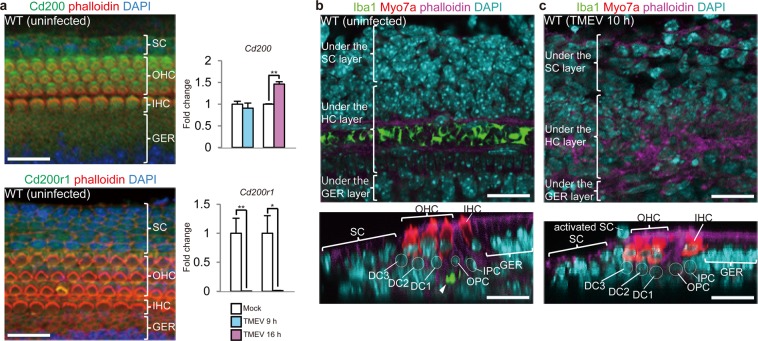
Both microglia and macrophages comprise the immune system in the cochlear sensory epithelium. (**a**) The Cd200-Cd200r1 system restricts microglial functions in the brain. Cd200 (green), the ligand of Cd200r1, was mainly expressed in HCs, but also weakly in SCs and GERCs. Cd200r1 (green) was expressed in SCs and GERCs. These findings indicate that Cd200-Cd200r1-specific intercellular signalling restricts SC/GERC functions to macrophages. During virus infection, *Cd200r1* mRNA was significantly downregulated (Mock 9 h: n = 3, TMEV 9 h: n = 4, Mock 16 h: n = 4, TMEV 16 h: n = 3), despite little change in *Cd200* expression (Mock 9 h: n = 3, TMEV 9 h: n = 4, Mock 16 h: n = 3, TMEV 16 h: n = 3) (*P < 0.05, **P < 0.01, *t*-test). These findings indicate that virus infection removes the Cd200-Cd200r1 restriction on SCs and GERCs, which induces their differentiation into macrophages. (**b**) In uninfected cochleae, Iba1-positive cells (green) were concentrically arranged beneath OPCs and DC1s, which might be cochlear resident microglia (arrowhead; outer PC: OPC, inner PC: IPC, DC1: first row of DCs, DC2: second row of DCs, DC3: third row of DCs). (**c**) Cochlear resident microglial cells were not observed in their original location during the virus infection. Scale bars, 20 µm. Error bars, standard errors.

Under steady-state conditions, microglial cell receptors, including CD200 receptor 1 (Cd200r1), interact with neural cell-surface ligands, resulting in the inhibition of microglial cell activity^32,33^. As shown in Fig. 4a, Cd200r1 was expressed in SCs and GERCs, and its ligand, cluster of differentiation 200 (Cd200), was mainly expressed in HCs, as well as in SCs and GERCs, suggesting that SCs and GERCs are inhibited by HCs and by each other. In response to virus infection, *Cd200r1* expression was suppressed (Fig. 4a, lower right), suggesting the CD200-CD200R-mediated suppression of macrophage-like function^32,33^ was inhibited by virus-infection similar to the microglia system. SCs and GERCs differ from the typical microglia that patrol the brain microenvironment in that SCs and GERCs tightly adhere to each other and to HCs. SCs are thought to primarily separate the endolymph and perilymph via the tight junction architecture, thereby maintaining the integrity of the sensory epithelium against mechanical stress from the vibrations transmitted by sound waves^34^. As potential macrophage-like cells, this architecture may suppress the shape and function of the SCs and GERCs (Fig. S1a). However, upon virus infection, the SCs and GERCs altered their shapes to become more macrophage-like and were able to migrate (Fig. S1b,c). To conduct a faint sound wave, only a minimal structure is required in the sensory epithelium. Therefore, SCs and GERCs might play diverse roles in sound conduction, homeostasis and immunity. In the present study, we analysed viral infection in cochlear sensory epithelia isolated from newborn mice. In the SHIELD (Shared Harvard Inner-ear Laboratory Database^35^) database, we found that genes associated with macrophages and inflammation that target pathogens were expressed in the SC fractions during development (embryonic day E16, postnatal days P0, P4 and P7; Fig. S3), suggesting that macrophage-like SCs also exist in adult mouse cochleae.

Interestingly, we identified a row of cells that expressed the common microglial marker Iba1^32,36^ under Deiters’ cells (DCs) and pillar cells (PCs)^32^ (Fig. 4b). However, these Iba1^+^ cells disappeared after viral infection (Fig. 4c), suggesting that they migrate in response to infection and function as typical microglial cells.

### SCs and GERCs phagocytose bacteria without cell migration

**Figure 5:**
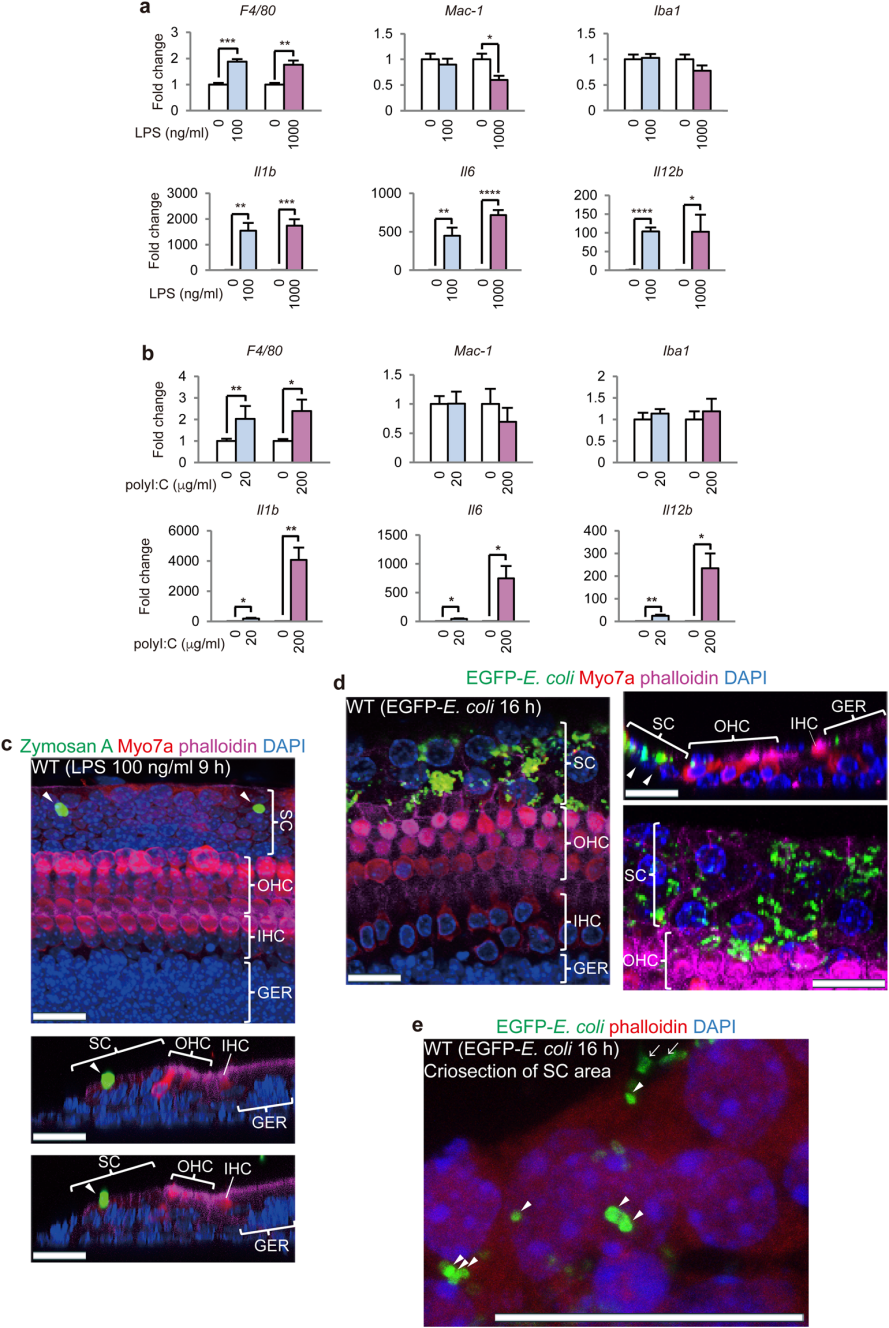
Phagocytosis of SCs and GERCs without migration. (**a**) qRT-PCR analysis of macrophage markers (*F4/80*, *Mac-1*, and *Iba1*) and M1 markers (*Il1b*, *Il6*, and *interleukin 12b* (*Il12b*)) after LPS treatment (time point, 9 h; *P < 0.05, **P < 0.01, ***P < 0.001, ****P < 0.0001, *t*-test; 0 ng/ml: n = 4, 100 ng/ml: n = 4, 0 ng/ml: n = 6, 1000 ng/ml: n = 4 for *Il12b*; 0 ng/ml: n = 4, 100 ng/ml: n = 4, 0 ng/ml: n = 4, 1000 ng/ml: n = 4 for the other genes). LPS-induced macrophage marker expression indicates that LPS stimulates SCs and GERCs as cochlea-resident macrophages. (**b**) qRT-PCR analysis of macrophage markers (*F4/80*, *Mac-1*, and *Iba1*) and M1 markers (*Il1b*, *Il6*, and *Il12b*) after poly I:C treatment (time point, 9 h; *P < 0.05, **P < 0.01, *t*-test; 0 µg/ml: n = 4, 20 µg/ml: n = 4, 0 µg/ml: n = 5, 200 µg/ml: n = 4 for *F4/80*; 0 µg/ml: n = 4, 20 µg/ml: n = 4, 0 µg/ml: n = 3, 200 µg/ml: n = 4 for the other genes). Poly I:C upregulated macrophage marker expression, indicating that poly I:C also activates SCs and GERCs as cochlea-resident macrophages. (**c**) SC phagocytosis was observed during LPS treatment. (**d**) Sets of immunostained whole mounts and sections showing EGFP-*E. coli* in the SCs (white arrowheads) during EGFP-*E. coli* infection. The lower right image of SCs also shows green signals in these cells, indicating SC phagocytosis of the bacteria. (**e**) The confirmation of SC phagocytosis of EGFP-*E. coli* using cryosections from the cochlear sensory epithelium after EGFP-*E. coli* infection. SC cryosections after EGFP-*E. coli* infection showing EGFP signals inside (arrowheads) and outside (arrows) the SCs. EGFP signals inside the SCs indicate phagocytosis of *E. coli* by the SCs, while EGFP signals outside the SCs indicate *E. coli* attachment to the SC surfaces. These findings show that SCs undergo phagocytosis during viral and bacterial infections, which strongly supports a role for SCs in mounting an innate immune response against microbes as macrophages. Scale bars, 20 µm. Error bars, standard errors.

To gain further insight into the mechanisms involved in the macrophage activation of SCs, we stimulated SCs with various pathogen-associated molecules known to activate macrophages. The induction of macrophage-associated markers and proinflammatory cytokines was observed following stimulation with lipopolysaccharide (LPS) and double-stranded (ds)RNA, both of which are recognised by Toll-like receptors^37^ (Fig. 5a,b and Fig. S4a–d). Moreover, the LPS-induced phagocytosis of SCs was observed (Fig. 5c), suggesting a protective role for SCs against pathogens other than viruses. Indeed, the SCs were able to phagocytose *Escherichia coli* in the absence of cell migration (Fig. 5d,e). Although *Cd200r1* expression was suppressed by LPS and dsRNA stimuli (Fig. S4e,f), the SCs and GERCs did not migrate (Fig. S4g,h). In fact, our cDNA microarray analysis revealed the suppression of many cell adhesion proteins following virus infection, but not after LPS stimulation (Fig. S4i).

### Role played by Irf5 in changing SCs into macrophage-like cells

**Figure 6:**
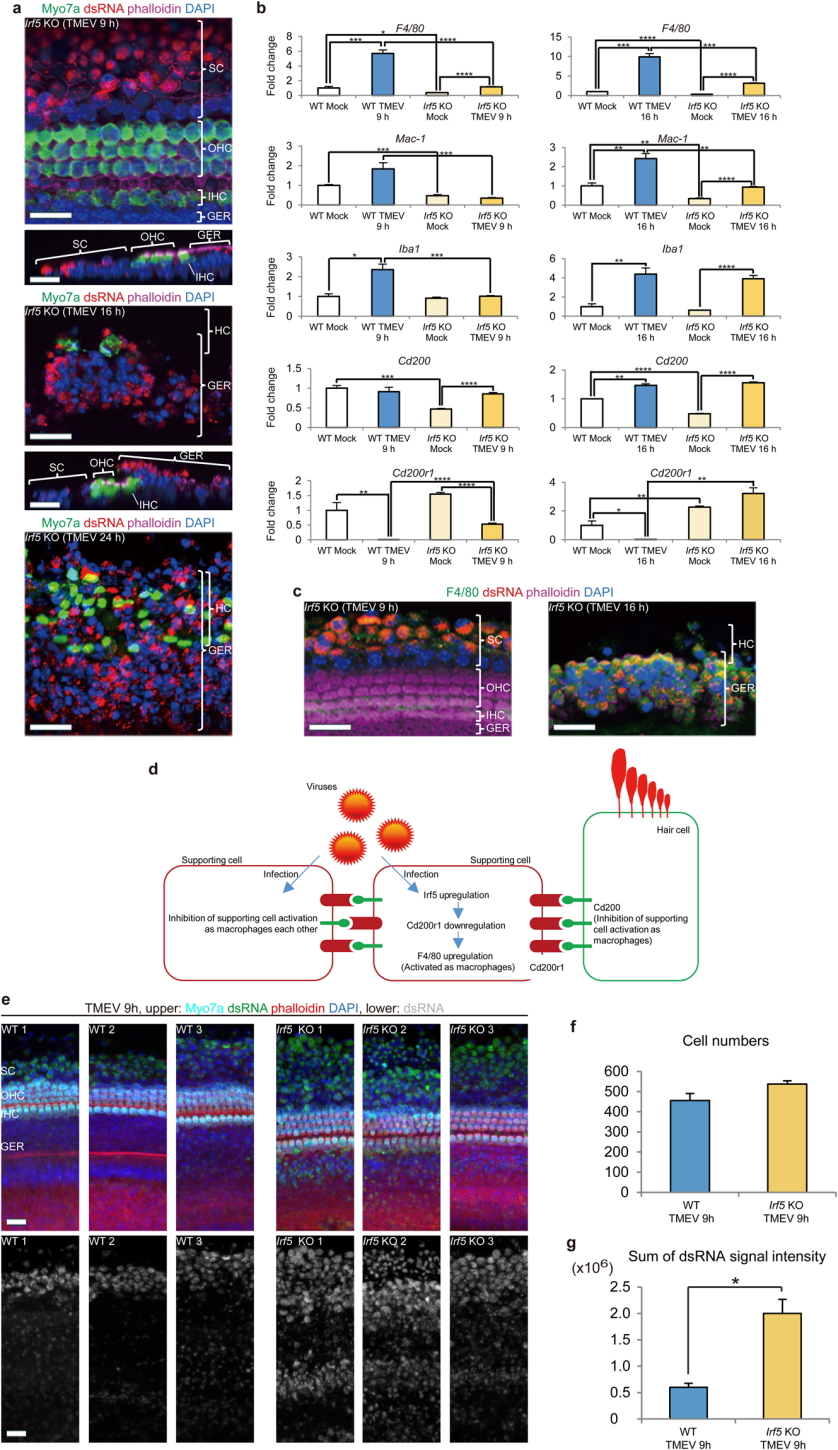
Irf5 is involved in differentiation of the SCs into macrophages via Cd200r1 downregulation during virus infection. (**a**) In *Irf5* KO mice, GERCs, but not SCs, migrated during the viral infection. (**b**) qRT-PCR analysis of *F4/80*, *Mac-1*, *Iba1*, *Cd200*, and *Cd200r1* (*P < 0.05, **P < 0.01, ***P < 0.001, ****P < 0.0001, *t*-test, *Irf5* KO Mock 9 h: n = 4, *Irf5* KO TMEV 9 h: n = 5, *Irf5* KO Mock 16 h: n = 4, *Irf5* KO TMEV 16 h: n = 4). Compared with WT mice during the virus infections, *F4/80* transcripts were fewer in the *Irf5* KO mice, *Cd200r1* transcripts were more in the *Irf5* KO mice, whereas *Cd200* transcripts in *Irf5* KO mice induced by the virus infection were similar to the levels in the WT mice. These findings show that Irf5 activates macrophages by regulating F4/80 expression through Cd200r1 inhibition. *Mac-1* expression in *Irf5* KO mice did not increase during virus infection compared with WT mice, but *Iba1* expression in *Irf5* KO mice was upregulated by virus infection to almost the same level as in the WT mice. This indicates that Iba1-positive cells are not regulated by Irf5. (**c**) F4/80 expression in *Irf5* KO mice increased in the GERCs but not in the SCs following virus infection, indicating that Irf5 regulates SC but not GERC differentiation into macrophages. (**d**) Scheme showing the intra- and inter-cellular signalling pathways involved in SC differentiation into macrophages. (**e**) Compared with WT samples where the SC ar**e**a is mainly infected with TMEV (dsRNA positive), *Irf5* KO samples show strong signal intensity of dsRNA over a broad area including the GER during virus infection. (**f**) There is no statistical difference in numbers of cells between WT (n = 3) and *Irf5* KO (n = 3) shown in (**e**) (*P* = 0.10412149, *t*-test). (**g**) *Irf5* KO samples (n = 3) show a significant increase in dsRNA signal intensity compared with WT samples (n = 3) (*P < 0.01, *t*-test), indicating the lack of SC conversion to macrophages accelerated virus infection in the cochlea. Scale bars, 20 µm. Error bars, standard errors.

In the sensory epithelium of *Irf5*^*−/−*^ mice where M1 macrophage polarization is suppressed^25,38^, induction of the genes encoding *F4/80*, *Mac-1* and *Iba1* macrophage markers, suppression of *Cd200r1* (Fig. 6b), and migration of the SCs in response to virus infection (Fig. 6a,c, compared to Fig. 2a) were all impaired. This indicates that virus-induced macrophage activation and SC migration are partly regulated by *Irf5* (Fig. 6d). Conversely, the virus-induced migration of GERCs was still observed in the *Irf5*^*−/−*^sensory epithelium (Fig. 6a,c). Moreover, *Irf5* knockout accelerated TMEV infection in cochlear sensory epithelia, suggesting that Irf5-mediated macrophage polarization is required for efficient protection against virus-infection (Fig. 6e–g). These results indicate that SCs and GERCs are distinct types of macrophage-like cells, and that different signalling mechanisms are required for macrophage activation and the migration of these cells.

## Discussion

The present study provides initial evidence supporting the macrophage-like nature of cochlear SCs and their potential importance in anti-viral and anti-bacterial defences in the inner ear. Many tissue-resident macrophages, especially microglia, arise from the embryonic precursors that take residence prior to birth and maintain a local position throughout adulthood^30,39^. It has been shown that the third DC row and inner PCs adjacent to HCs (Fig. 4b,c) act as HC progenitors following injury^40^, suggesting that their origin is the same as that of HCs. Like HCs, SCs are also considered to be ectoderm cells, but our results suggest the possibility that some SCs, including Hensen’s cells and Claudius’ cells, have a mesodermal cell origin. Indeed, virus- or LPS-stimulated SCs displayed characteristics typical of macrophages, as determined by our analysis of macrophage marker protein expression, transcriptional profiles, and phagocyte activity and morphology, suggesting that SCs are tissue-resident macrophages. Moreover, under our experimental conditions, isolated sensory epithelium contained rows of Hensen’s cells and 4–6 rows of Claudius’ cells, both of which were infected with the virus and migrated with increased expressions of macrophage markers including F4/80, Irf5, and Jmjd3. This suggested that Hensen’s cells and Claudius’ cells function as macrophages. A notable difference between SCs and other resident macrophages, especially microglia, is that uninfected SCs show no typical features of macrophages, particularly in terms of their morphology, and are anchored by tight junctions in their resting state. However, it has been shown that tight junction proteins are expressed in lung epithelium macrophages^41^.

Here, we analysed viral infections in isolated cochlear sensory epithelia from the soft temporal bones of newborn mice, because it is difficult to obtain viable organs of Corti that have maintained their tissue structures from the temporal bones of adult mice. Therefore, our analyses have some limitations. During development, cochlear extension and coiling continue until approximately E19 or P0, by which time the duct has reached its mature shape. At the tissue level, cellular differentiation within the prosensory domain is observable in the mid-basal region of the cochlea between E14 and E15. Developing OHCs are observable by E15 to E16, and developing SCs become morphologically distinct around the same time. By E17, HCs and SCs along the length of the cochlear duct have become arranged into the characteristic pattern for the organ of Corti^42,43^. Although full cochlear function takes some time to complete after birth, it is considered that the cellular differentiation and organization of the organ of Corti is completed before birth. This is supported by our findings that show that the genes associated with macrophages and inflammation were expressed in the cochlear SC fractions after birth (P0, P4 and P7; Fig. S3). Therefore, although the neonatal organ of Corti is in a different functional state when compared with the adult mouse, our experimental results were at least obtained from tissues with the same cell composition as the adult mouse. Therefore, in the same way that microglial cells arise from embryonic precursors, it is conceivable that embryonic-stage macrophage-like cells play a role in infection prevention in the adult mouse.

The stria vascularis of the inner ear is a highly specialised capillary network that controls exchanges between blood and the intrastitial cochlea space, and is enriched in tight junctions^1^. In a murine model of cytomegalovirus (CMV)-induced hearing loss, cells expressing a CMV-encoded protein were detected in the stria vascularis^44^, suggesting that viruses invade the inner ear endolymph from the blood flow through the stria vascularis. If pathogens can invade the first line of defence in the stria vascularis, there is no immune protection mechanism in the endolymph because immune cells do not circulate in this region. In the audiosensory organ, massive cell movement might cause ectopic sound waves; therefore, newly identified anchored-phagocyte systems that surround HCs are useful for providing a silent protection system in audiosensory organs. However, with viral infections, virus-infected cell elimination may occur by inhibiting the expression of cell adhesion molecules via the MAVS-signalling cascade, and following the induction of virus-induced apoptosis in the infected cells. It is not currently clear that such cell migration is observed in the adult mouse. It is possible that SC migration in response to viral infection results in electrical leakage and electrolyte imbalance, and that this imbalance impairs cochlear function^45^. Thus, although this system works well for low-level viral infections, it may not be able to protect HCs from severe viral infections. In addition, activation of the macrophage-like function of SCs was followed by that of GERCs. Considering GERCs were relatively polarised to an anti-inflammatory M2 phenotype, these delayed activated GERCs might suppress inflammatory reactions and promote tissue repair in the organ of Corti.

Unlike viral infections, in bacterial infections, the expression of cell adhesion molecules in HCs does not change and bacterial phagocytosis occurs. This phenomenon seems to be a beneficial system to eliminate bacteria while causing minimal tissue damage. However, our current results only showed the phagocytic activity of SCs. Further analyses are required to elucidate the protective role of SCs against bacterial infection, using clinically known bacteria that cause inner ear infection. In addition, we showed that microglia-like Iba1-positive cells were present under the HC layer. These cells disappeared after viral infection, but we have no direct evidence that they migrated to the site of viral infection, at present. Moreover, it is still unclear whether these cells exist in adult mice. However, very few macrophages are present in the sensory epithelium^13^. Therefore, if an infection occurs near HCs, these pre-existing macrophages might traffic to the infected area to protect HCs from the pathogen in cooperation with the SCs. These double-defence mechanisms may protect audiosensory organs from viruses and other pathogens.

## Methods

### Experimental animals

Postnatal day 2 (sex: unknown) ICR mice (SLC), *Ifnar1* null mice (B&K Universal), and *Irf5* null mice^38^ were used in this study. All experimental procedures were performed in accordance with the NIH Guide for the Care and Use of Laboratory Animals. The Animal Research Committee of Nippon Medical School approved all experimental protocols.

### Preparation and treatment of cochlear sensory epithelium explant cultures

Cochlear sensory epithelia were resected and cultured as previously described^21^. All experiments began after an overnight incubation of each cochlea with 300 µl medium to stabilize the explants. Each cochlea was then transferred to 400 µl medium containing 3.0 × 10^7^ pfu/ml TMEV, which is an RNA virus. TMEV (GDVII strain) was propagated from viral cDNA and BHK21 cells^46^. Cultures were maintained for 9–12 h until TMEV began to infect the SCs. At 16–21 h, TMEV began to infect the GERCs and HC death was observed. HC death was almost completed by 24 h. To determine the characteristics of macrophage marker-positive SCs and GERCs, we analysed the phagocytosis of these cells using Zymosan A (Invitrogen), chemically or heat-killed *Saccharomyces cerevisiae* conjugated with Alexa Fluor 488. At 9 or 19 h after TMEV or lipopolysaccharide (LPS) incubation, Zymosan A (16 µl) diluted 1:100 with phosphate-buffered saline (PBS) was added to the medium for 1 h. To determine whether SCs acquired phagocytosis capabilities during virus and bacterial infection, we used EGFP-*E. coli*. EGFP cDNA was cloned into a GST expression vector, pGEX6P-1 (GE Healthcare). The *E. coli* strain, BL21(DE3)-RIL (Agilent Technologies) was transformed with the pGEX6P-1-EGFP plasmid and shaking-cultured in LB medium at 37 °C, 200 rpm for 2 h. EGFP protein expression was induced by the addition of 1 mM IPTG for 1.5 h. After shaking-culture, LB medium with transformed *E. coli* was diluted 20 times with Dulbecco’s modified Eagle’s medium (DMEM) without any antibiotics. Cochlear sensory epithelia were cultured in this medium for 16 h. To evaluate the influence of retinoic acid inducible gene-I (RIG-I)-like receptor (RLR) stimulation by virus infection and toll-like receptor (TLR) stimulation, we used LPS and poly I:C. The explants were transferred to medium containing 100 ng/ml or 1000 ng/ml LPS (Sigma-Aldrich) for up to 24 h or 20 µg/ml or 200 µg/ml poly I:C (Sigma-Aldrich) for up to 48 h. To determine whether RNA virus and DNA virus induced SC and GERC activation as macrophages, we used herpes simplex virus 1 (HSV-1) carrying GFP (YK333)^47^. This recombinant virus possesses a complete set of viral genes and exhibits the same growth properties as the wild-type virus. In cells infected with GFP-HSV-1, GFP expression is driven by the host early growth response 1 (Egr-1) promoter^48^. GFP-HSV-1 was produced by Vero cells^49^ in the absence of antibiotics to exclude the possibility that administered antibiotics could injure cochleae. The explants were infected with 1.0 × 10^7^ pfu/ml GFP-HSV-1 for up to 24 h. The common downstream molecule after TMEV and HSV-1 infection is interferon-β promoter stimulator 1 (IPS-1)^47^, which is not upregulated by LPS or poly I:C stimulation. We hypothesized that IPS-1 is a key molecule involved in SC migration during virus infection. Based on this, we transfected SCs with IPS-1 lentivirus vector. Mouse IPS-1 cDNA was synthesized from murine embryonic fibroblasts by RT-PCR. The N-terminal 2 human influenza hemagglutinin (HA)-tagged mouse IPS-1 (2HA-IPS-1) was constructed by cloning the above IPS-1 cDNA into the pCDH-EF1-MCS-IRES-Puro vector (System Biosciences). The constructs were transfected into 293 T packaging cells along with the packaging plasmids. Initially, the cochlear explants were incubated for 24 h in the above lentivirus-containing supernatants diluted two times with DMEM, to which 8 µg/ml polybrene was added, and cultured for the next 48 h in the supernatants prepared anew in the same way.

### Immunohistochemistry

For whole-mount immunohistochemistry, samples were fixed at room temperature (RT) for 15 min in 4% paraformaldehyde in 0.1 M phosphate buffer (pH 7.4), then rinsed with PBS. Samples for cryosections were fixed at RT for 15 min in 4% paraformaldehyde in 0.1 M phosphate buffer (pH 7.4). After rinsing with PBS, they were embedded in OCT compound (Tissue Tek), and sections including planes around the centre of the concentric circle were prepared. All specimens were incubated in blocking solution at RT for 30 min in 10% goat serum with 0.2% Triton X-100 for all antibodies except Cd200r1 and HA or for 15 min in 0.2% Triton X-100 and 15 min in 1% BSA in 0.2% Triton X-100 when co-labelled with Cd200r1 or HA. The primary antibodies used in this study were as follows: rabbit polyclonal anti-Myosin VIIA (Myo7a) (25–6790; 1:1000) from Proteus Biosciences, rabbit polyclonal anti-Ifnar1 (ab62693; 1:50) from Abcam, rabbit polyclonal anti-Ifnar2 (orb100572; 1:100) from Biorbyt, mouse monoclonal anti-double-stranded RNA (dsRNA) (J2; 1:800, K1; 1:2000) from English & Scientific Consulting, rabbit polyclonal anti-Jmjd3 (ab38113; 5 µg/ml) from Abcam, mouse monoclonal anti-Irf5 (10T1; 1:50) from Abcam, rat monoclonal anti-F4/80 (A3-1; 1:50) from AbD Serotec, rabbit polyclonal anti-Cd200 (ab203887; 1:500) from Abcam, goat polyclonal anti-Cd200r1 (AF2554; 5 µg/ml) from R&D Systems, rat monoclonal anti-GFP (GF090R; 1:500) from Nacalai Tesque, and rat monoclonal anti-HA (3F10; 1:500) from Roche. Actin filaments were visualized with Alexa 594- or 633-labelled phalloidin (1:100, Invitrogen). The primary antibodies were visualized with Alexa 488- or 546-conjugated anti-rabbit, anti-mouse, or anti-rat goat IgG or Alexa 488-conjugated anti-goat donkey IgG (1:1000, Invitrogen). Samples with nuclear staining were then incubated in PBS containing 1 μg/ml DAPI (Invitrogen). Fluorescent images were captured using an Olympus FV1200 confocal microscope. The computational section images were reconstructed by FV10-ASW (Olympus) after capturing images every 0.2–0.5 µm. The whole-mount images of HCs were obtained by superposing images from the bottom to the top of HCs after capturing images every 0.5 µm. The whole-mount images of virally infected, activated SCs or GERCs were obtained from single slices or several slices overlapped at the SC or GERC level after capturing images every 0.5 µm.

### Cell counts

The numbers of dsRNA and Myo7a double-positive inner and outer HCs in the sensory epithelium were counted in one high-power field (318 × 318 µm) in the basal- to mid-turn of each explant. As described above, we estimated from the basal- to mid-turn of the cochlea, because Hensen’s cells and Claudius’ cells in the apical turn overlapped with the inner side of the basal-turn.

### Electron microscopy

Electron microscopy analysis was performed as described previously^50,51^, with slight modification. Mouse inner ears were observed by transmission and scanning electron microscopy (SEM). The specimens were fixed with 2% glutaraldehyde in 0.1 M phosphate buffer (pH 7.4) for 60 min, washed five times in 0.1 M phosphate buffer, and post-fixed with 1% osmium tetroxide for 60 min at 4 °C. For transmission electron microscopy (TEM), the fixed inner ears were dehydrated with a graded ethanol series, and embedded in EponA2 according to the conventional method. Thin sections were cut with a diamond knife, stained with uranyl acetate and lead citrate, and examined with a JEM-1010 transmission electron microscope (JEOL) at an accelerating voltage of 80 kV. For SEM, the fixed inner ears were dehydrated with a graded ethanol series and then freeze-dried (Hitachi ES-2020, Hitachi) in *t*-butyl alcohol. After coating with osmium tetroxide (approximately 10 nm thick) using an osmium plasma coater (NL-OPC80, Nippon Laser & Electronics Lab.), the specimens were examined with a Hitachi S-4500 field emission SEM at an accelerating voltage of 10 kV.

### RNA extraction, qRT-PCR, and microarray

For qRT-PCR, the RNeasy Micro Kit (Qiagen) was used to extract total RNA from three cochlear sensory epithelia, which were cultivated under the following conditions for 9 or 16 h: DMEM alone; DMEM with TMEV; DMEM with LPS; and DMEM with poly I:C, respectively. cDNA was synthesized from DNase-treated total RNA using the PrimeScript RT reagent Kit (Takara Bio). Synthesized cDNA was subsequently mixed with TaqMan Universal PCR Master Mix (Applied Biosystems) in the presence of commercial TaqMan primer-probe sets of interest (Applied Biosystems). Real-time PCR quantification was performed using the ABI StepOnePlus Real-Time PCR System (Applied Biosystems). All reactions were performed in triplicate. The relative mRNA amounts were calculated using the ΔΔCt method. For the invariant control, we used *actin beta* (*Actb*). For microarray, total RNA was extracted from nine mock explants, nine explants treated with 1000 ng/ml LPS for 16 h, or 14 explants infected with TMEV for 16 h using the RNeasy Micro Kit. TMEV infection for 16 h induced cochlear cell death in HCs and SCs; therefore, a greater number of TMEV-infected cochleae was needed compared with the mock and LPS-treated cochleae to equalize the amount of total RNA. Filgen (a biological technical service company) performed the microarray analysis using the GeneChip Mouse Gene 2.0 ST Array (Affymetrix).

### Live imaging

TMEV-infected cochleae were cultivated on a collagen-coated 35-mm glass-bottom dish (MatTek) for live imaging. For visualizing activated SCs and GERCs as macrophages, a Leica SP8 confocal laser-scanning microscope equipped with a HC PL APO 63×/1.40 CS2 oil objective was used. Images were captured every 5 min, from 8 to 25 h after TMEV infection, using a 488-nm argon laser with 10% intensity. While capturing images, the explant was maintained in a CO_2_ (5%) stage-top incubator (INUBG2F-GSI2, Tokai Hit) at 37 °C. The movies were constructed using Leica LAS AF software (9–14 h and 14–21 h, respectively).

### Statistical analysis

The data are expressed as the mean ± standard error. Unpaired *t*-tests were used for Myo7a and dsRNA double-positive HCs, in which *P*-values <0.05 were considered statistically significant. Unpaired *t*-tests were used for cell numbers or sum of dsRNA signal intensity in WT and *Irf5* KO cochleae infected with TMEV for 9 h, in which *P*-values <0.05 were considered statistically significant. In all qRT-PCR analyses, unpaired *t*-tests were used, in which *P*-values <0.05 were considered statistically significant.

## Supplementary information

Supplementary Information.

Supplementary Video 1.

Supplementary Video 2.
