# Supplementary material for: Cochlear supporting cells function as macrophage-like cells and protect audiosensory receptor hair cells from pathogens
Source: Sci Rep. 2020 Apr 21;10:6740. doi: 10.1038/s41598-020-63654-9 (PMC7174420; doi:10.1038/s41598-020-63654-9)
Supplement: Supplementary file 1 — Supplementary Information. [file 41598_2020_63654_MOESM1_ESM.pdf]

Supplementary information

Title: Cochlear supporting cells function as macrophage-like cells and protect audiosensory receptor hair cells from pathogens

Authors: Yushi Hayashi[1], Hidenori Suzuki[2], Wataru Nakajima[1], Ikuno Uehara[1], Atsuko Tanimura[1], Toshiki Himeda[3], Satoshi Koike[4], Tatsuya Katsuno[5], Shin-ichiro Kitajiri[5], Naoto Koyanagi[6], Yasushi Kawaguchi[6], Koji Onomoto[7], Hiroki Kato[8], Mitsutoshi Yoneyama[7], Takashi Fujita[8], & Nobuyuki Tanaka[1, *]

[1]Department of Molecular Oncology, Institute for Advanced Medical Sciences, Nippon Medical School, Tokyo, Japan. [2]Division of Morphological and Biomolecular Research, Nippon Medical School, Tokyo, Japan. [3]Department of Microbiology, Kanazawa Medical University School of Medicine, Ishikawa, Japan. [4]Neurovirology Project, Tokyo Metropolitan Institute of Medical Science, Tokyo, Japan. [5]Department of Otolaryngology, Head and Neck Surgery, Kyoto University, Kyoto, Japan. [6]Division of Molecular Virology, Department of Microbiology and Immunology, The Institute of Medical Science, The University of Tokyo, Tokyo, Japan. [7]Division of Molecular Immunology, Medical Mycology Research Center, Chiba University, Chiba, Japan. [8]Laboratory of Molecular Genetics, Institute for Virus Research, Kyoto University, Kyoto, Japan.

[*]corresponding author, email: nobuta@nms.ac.jp

Supplementary Figure Legends

Figure S1. SCs and GERCs are activated as macrophages and start to migrate to HCs via IPS-1 upregulation following virus infection. (**a**) SEM of the uninfected cochlear surface. All cells were fixed in the sensory epithelium and no cells moved onto the epithelial surface. (**b**) Innumerable activated SCs and GERCs moved as macrophages onto the epithelial surface and migrated to the HC layer (21 h, SEM). Most HCs died and very few deformed stereocilia of the HCs remained (red arrowheads). (**c**) High-magnification image of SCs activated on the HC layer as macrophages with the formation of membrane protrusions and ruffles (24 h, SEM), whose shapes changed from steady-state SCs and GERCs embedded and fixed in the cochlear sensory epithelium. (**d**) GFP-lentivirus vector infected SCs, such as Hensen's cells and Claudius' cells (white arrowhead), revealing that this vector effectively transfers genes to SCs. (**e**) SCs infected with the 2HA-IPS-1 lentivirus vector migrated to the sensory epithelium surface (white arrowheads), indicating that IPS-1 upregulation is responsible for SC migration following virus infection. Scale bars in (**a–c**), 5 μm and in (**d,e**), 20 μm.

Figure S2. Macrophage marker expression is induced by virus infection. Microarray analysis revealed that many positive regulators of macrophage chemotaxis including chemokines and some macrophage markers were upregulated, and GATA binding protein 2 (*Gata2*), a negative regulator of macrophage differentiation, was downregulated following virus infection (16 h), as compared with mock and LPS treatment (16 h). Red, relative upregulation; green, relative downregulation; yellow, no change. These data reveal the strong drive for SCs and GERCs to

change into macrophages upon viral infection.

Figure S3. Expression-level changes for genes correlated with macrophage activity and anti-pathogen inflammation during development. (**a–c**) SHARED HARVARD INNER-EAR LABORATORY DATABASE (SHIELD, https://shield.hms.harvard.edu/index.html) is a resource for RNAseq data sets for HCs (GFP-positive cells) and their surrounding cells including SCs (GFP-negative cells) at E16, P0, P4 and P7. Here, we drew macrophage markers (**a**) toll-like receptor 3 (*Tlr3*), toll-like receptor 4 (*Tlr4*) (**b**) and *Cd200* and *Cd200r1* (**c**) from this database. The expression levels of these genes in SCs were upregulated or unchanged during maturation of the cochlear sensory epithelium while those in the HCs decreased or remained at low levels, indicating that the anti-microbe defence system used by the SCs is sustained until adulthood.

Figure S4. LPS or poly I:C administration upregulates macrophage markers but does not induce SC and GERC migration. Both removal of inhibition by Cd200-Cd200r1 and downregulation of cell adhesion molecule expression are responsible for SC and GERC migration as macrophages following virus infection. (**a,b**) qRT-PCR analysis of M1 markers (**a**) and M2 markers (**b**) after LPS treatment (time point, 9 h; $*P < 0.05$, $**P < 0.01$, $***P < 0.001$, $****P < 0.0001$, *t*-test; 0 ng/ml: n = 4, 100 ng/ml: n = 4, 0 ng/ml: n = 4, 1000 ng/ml: n = 4). LPS-induced M1 and M2 marker expression indicates that LPS stimulates SCs and GERCs as cochlea-resident macrophages. (**c,d**) qRT-PCR analysis of M1 markers (**c**) and M2 markers (**d**) after poly I:C treatment (time point, 9 h; $*P < 0.05$, $**P < 0.01$, $****P < 0.0001$, *t*-test; 0 µg/ml: n = 4, 20

μg/ml: n = 4, 0 μg/ml: n = 3, 200 μg/ml: n = 4). Poly I:C upregulated M1 and M2 marker expression, indicating that poly I:C also activates SCs and GERCs as cochlea-resident macrophages. (**e,f**) qRT-PCR analysis after LPS (**e**) (0 ng/ml: n = 4, 100 ng/ml: n = 4, 0 ng/ml: n = 4, 1000 ng/ml: n = 4) or poly I:C (**f**) (0 μg/ml: n = 4, 20 μg/ml: n = 4, 0 μg/ml: n = 3, 200 μg/ml: n = 4) treatment of the Cd200-Cd200r1 system (time point, 9 h; $*P < 0.05$, $**P < 0.01$, $***P < 0.0001$, *t*-test). (**g,h**) LPS treatment did not induce SC and GERC migration to HCs (**g**). Poly I:C treatment did not induce SC and GERC migration to the HC layer (**h**). These data indicate that not only removal of inhibition by the Cd200-Cd200r1 system but other mechanisms also operate for SC and GERC migration as macrophages following virus infection. (**i**) Microarray analysis revealed the decreased expression of many cell adhesion molecules following virus infection (16 h) compared with mock and LPS treatment (16 h), indicating that downregulation of cell adhesion molecules is a necessary condition for embedded and fixed SCs and GERCs in the epithelium to move and migrate. Red, relative upregulation; green, relative downregulation; yellow, no change in regulation. Scale bars, 20 μm. Error bars, standard errors.

Video Legends

Supplementary Video 1. Live imaging of SCs migrating to the HC layer during virus infection. TMEV-infected SCs migrate towards the HC layer (9–14 h). Hensen's cells and Claudius' cells migrate to HCs (moving downwards in the display) with hairs (stereocilia) that look like an inverted V in red (pseudocolour). The inverted V-shaped stereocilia degenerate and collapse

when the SCs touch them (the pseudocoloured structure in red disappears), indicating that activated SCs function as macrophages and induce HC death. This video was constructed using Leica LAS X software, of which URL is https://www.leica-microsystems.com/company/news/news-details/article/pharma-software-free-download-of-las-x/.

Supplementary Video 2. Live imaging of GERCs migrating to the HC layer during virus infection. TMEV-infected GERCs migrating towards the HC layer (14–21 h). Numerous GERCs are spread over the HC layer (moving upwards in the display) over time.

Figure S1

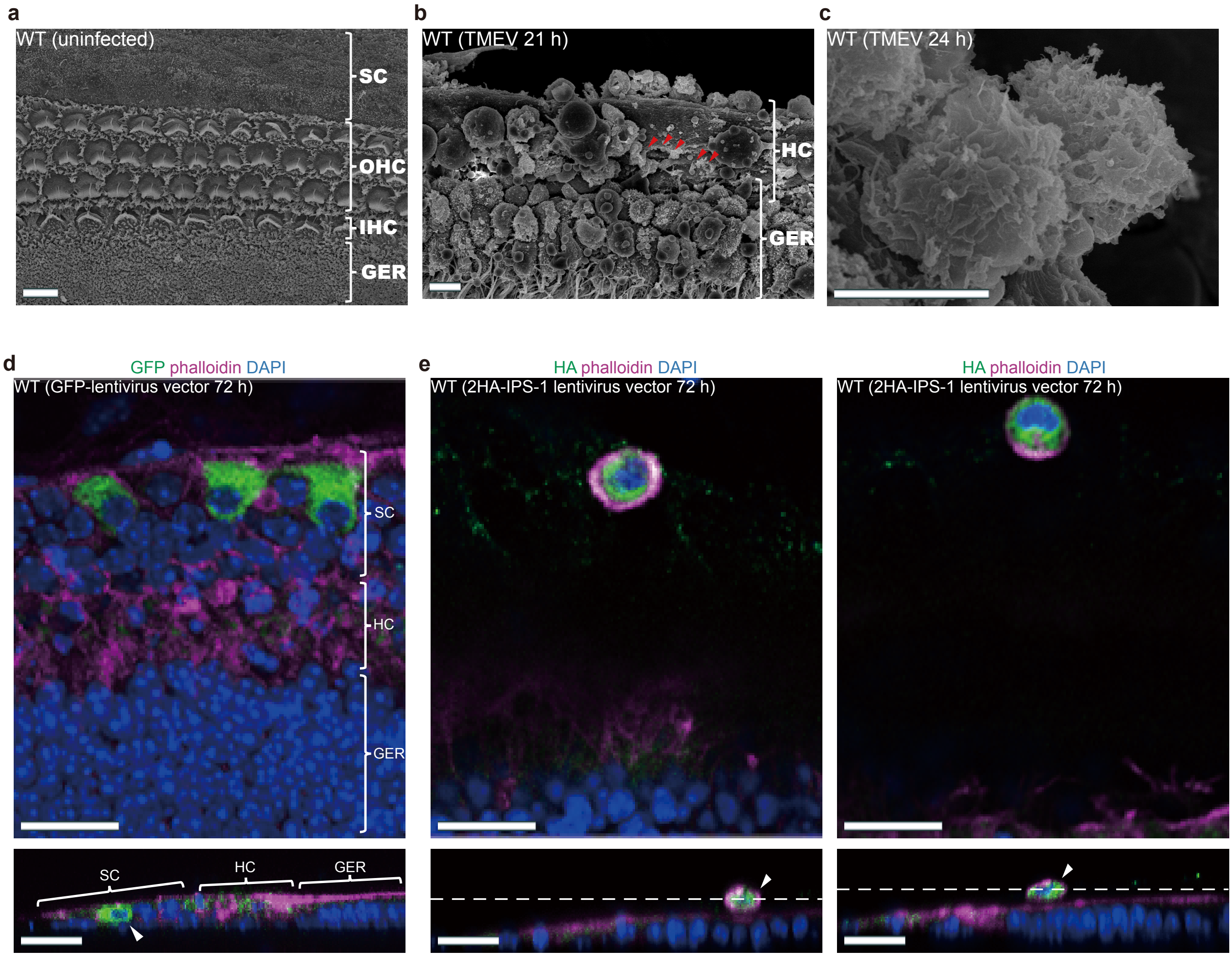
a
WT (uninfected)
SC
OHC
IHC
GER
b
WT (TMEV 21 h)
HC
GER
c
WT (TMEV 24 h)
d
GFP phalloidin DAPI
WT (GFP-lentivirus vector 72 h)
SC
HC
GER
e
HA phalloidin DAPI
WT (2HA-IPS-1 lentivirus vector 72 h)
HA phalloidin DAPI
WT (2HA-IPS-1 lentivirus vector 72 h)

Figure S2

	Mock	LPS	TMEV
Ccl3	1	0.40	1.70
Ccl5	1	0.32	1.28
Ccl12	1	1.07	3.67
Cxcl9	1	0.95	3.15
Cxcl10	1	0.38	10.61
Cxcl11	1	0.87	13.46
C3ar1	1	1.32	2.22
Thbs1	1	0.71	1.92
Cd52	1	0.85	2.02
Cd72	1	1.08	3.55
Cd274	1	0.91	10.52
Stat1	1	0.74	6.87
Macro	1	0.88	2.27
Gata2	1	1.04	0.40

★ positive regulator of macrophage chemotaxis

★ macrophage marker

☆ negative regulator of macrophage differentiation

Figure S3

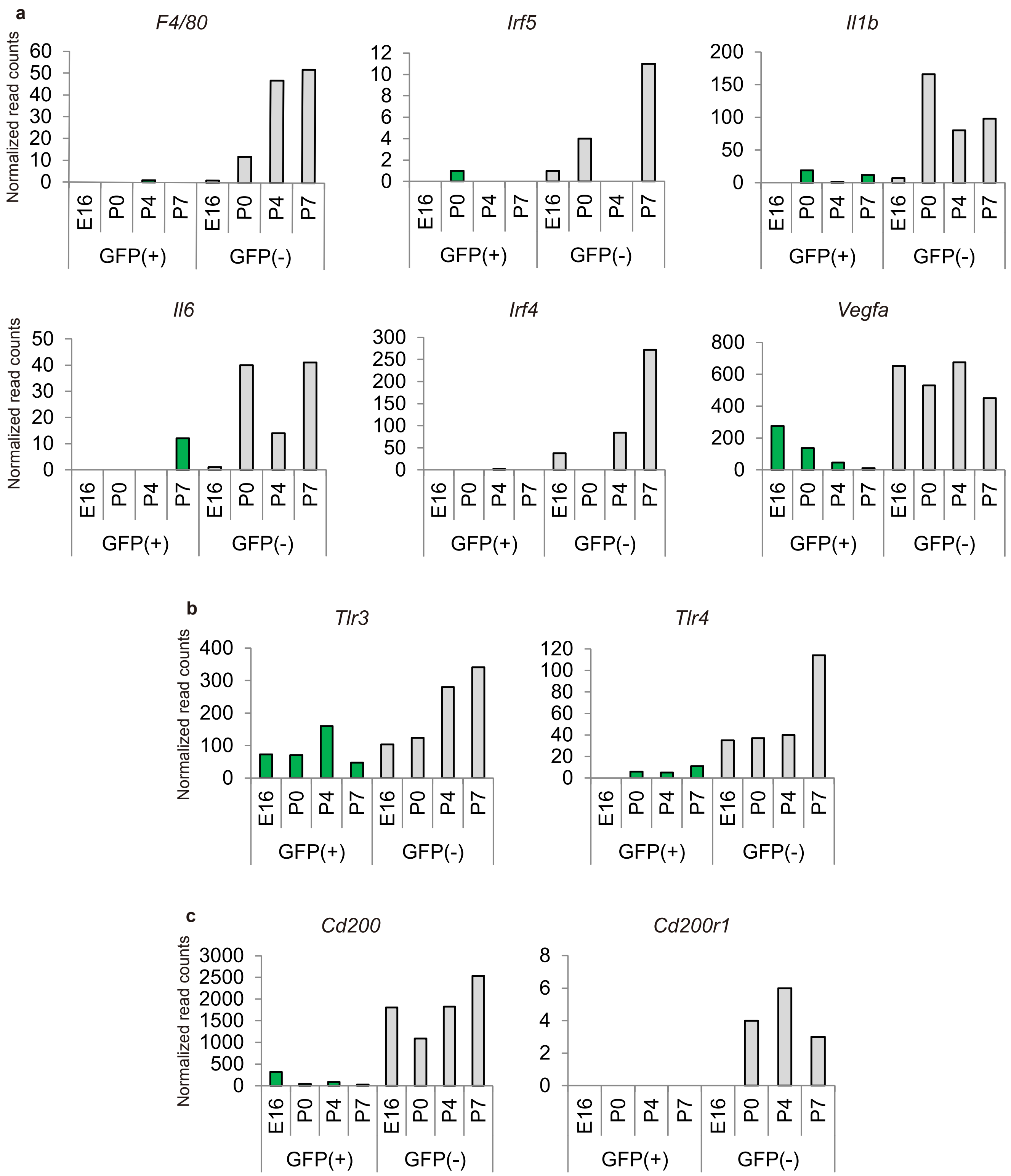
a
F4/80
Irf5
Il1b
Il6
Irf4
Vegfa
b
Tlr3
Tlr4
c
Cd200
Cd200r1
Normalized read counts
E16
P0
P4
P7
GFP(+)
GFP(-)

Figure S4

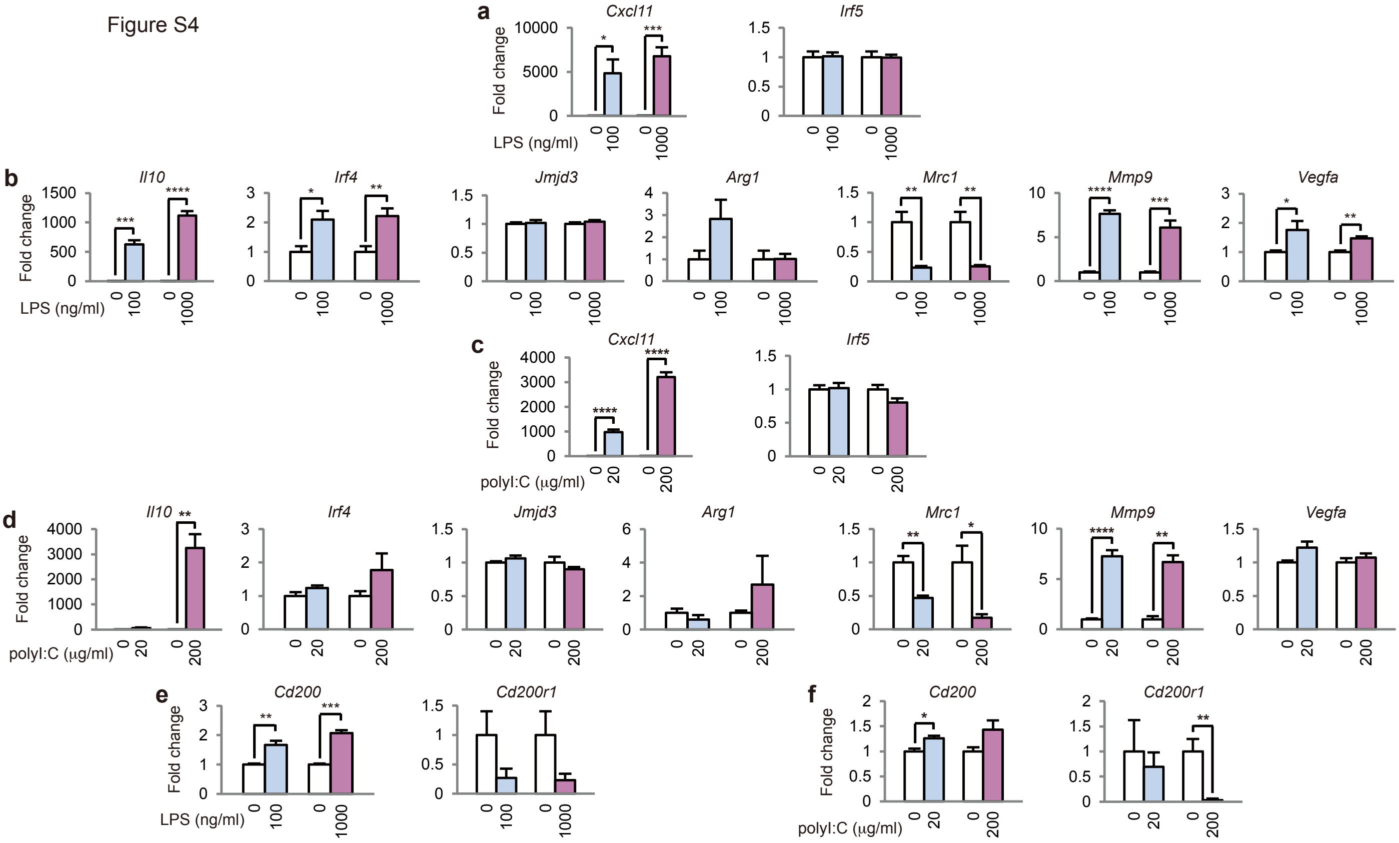

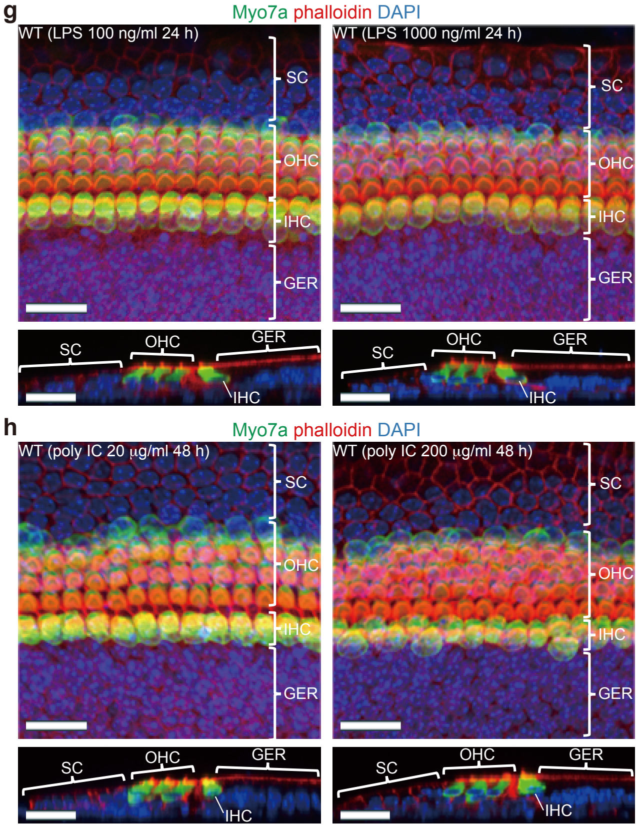

i

	Mock	LPS	TMEV
Itga1	1	1.29	0.44
Itgbl1	1	1.31	0.31
Itga10	1	1.30	0.36
Gjc3	1	0.80	0.29
Cdh6	1	1.29	0.16
Cdh10	1	1.12	0.30
Cdh18	1	0.70	0.33
Cdh19	1	1.63	0.40
Pcdh17	1	1.29	0.46
Pcdh18	1	1.07	0.23
Pcdhb18	1	0.79	0.38
Pcdhb19	1	1.00	0.44
Cldn11	1	0.99	0.33
Fn1	1	1.00	0.21
Adamts12	1	0.79	0.18
Jam2	1	1.03	0.38
Mpz	1	1.06	0.42
